# Polarised VEGFA Signalling at Vascular Blood–Neural Barriers

**DOI:** 10.3390/ijms19051378

**Published:** 2018-05-05

**Authors:** Silvia Dragoni, Patric Turowski

**Affiliations:** Institute of Ophthalmology, University College London, 11-43 Bath Street, London EC1V 9EL, UK; s.dragoni@ucl.ac.uk

**Keywords:** VEGF, endothelial cells, polarity

## Abstract

At blood–neural barriers, endothelial VEGFA signalling is highly polarised, with entirely different responses being triggered by luminal or abluminal stimulation. These recent findings were made in a field which is still in its mechanistic infancy. For a long time, endothelial polarity has intuitively been presumed, and likened to that of epithelial cells, but rarely demonstrated. In the cerebral and the retinal microvasculature, the uneven distribution of VEGF receptors 1 and 2, with the former predominant on the luminal and the latter on the abluminal face of the endothelium, leads to a completely polarised signalling response to VEGFA. Luminal VEGFA activates VEGFR1 homodimers and AKT, leading to a cytoprotective response, whilst abluminal VEGFA induces vascular leakage via VEGFR2 homodimers and p38. Whilst these findings do not provide a complete picture of VEGFA signalling in the microvasculature—there are still unclear roles for heterodimeric receptor complexes as well as co-receptors—they provide essential insight into the adaptation of vascular systems to environmental cues that are naturally different, depending on whether they are present on the blood or tissue side. Importantly, sided responses are not only restricted to VEGFA, but exist for other important vasoactive agents.

## 1. Endothelial Cell Polarity

**Figure 1:**
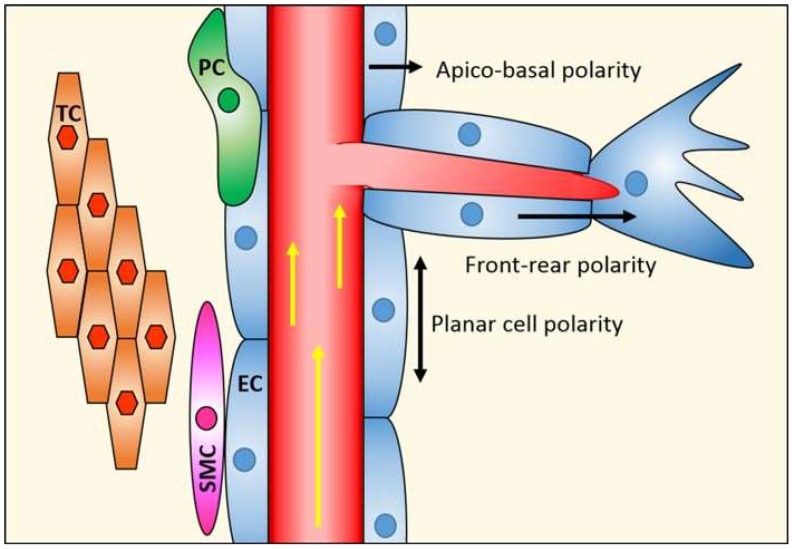
Vascular endothelial polarity. EC, endothelial cells; PC, pericytes; SMC, smooth muscle cells; TC, tissue cells.

Cell polarity arises through the asymmetric distribution of proteins, lipids, and nucleic acids between at least two poles of a cell. It plays a key role in intracellular transport, cell division, differentiation, cell movement, and morphogenesis [1,2]. Cells acquire particular types of polarity in order to perform specialised cell functions [3]. The ability to polarise is an intrinsic property of all cells, and is key for the development of multicellular organisms, as well as for maintaining tissue homeostasis. By contrast, loss of cell polarity accompanies increased cell proliferation, disorganisation, and tumourigenesis [4]. The molecular mechanisms underlying cell polarity are very well described in epithelial cells [5,6]. Endothelial cell (EC) polarity, despite being intuitively presumed, has not been investigated until recently, and its molecular framework is still largely unexplored [7]. The reason for this lag in understanding of endothelial polarity is undoubtedly the greater difficulty in separating, biochemically or microscopically, the basal and apical domains of these cells, which are many times thinner than epithelial cells (100 nm vs. several μm). However, it is now recognised that the endothelium can adopt three forms of polarity (Figure 1). Similar to the epithelium, endothelial monolayers display an apicobasal polarity with distinct luminal and abluminal domain facing the blood and the tissue, respectively [4]. In addition, the endothelium also exhibits features of planar cell polarity, where cells organise in the plane orthogonal to the apical–basal axis. Here, shear stress through blood flow provides important cues that polarise the EC cytoskeleton relative to flow [8]. Finally, during sprouting angiogenesis, tip cells display front–rear polarity akin to the leading and trailing edge described for migrating leukocytes [9,10].

Establishment of polarity should be viewed as the integration of the cell to extracellular cues and the formation of functional cortical domains adapted to the polarised environment. These domains then transmit the polarity to the rest of the cell, by regulating the organisation of the cytoskeleton and membrane trafficking system [11,12]. Thus, it is not surprising that at least some of the molecular pathways regulating and organising apicobasal and planar polarity share the same key regulators. For instance, WNT signalling, which is key to the planar cell polarity program in most cell types, is also instrumental for apicobasal polarity in brain ECs [13].

Endothelial polarity manifests itself in many ways. The apical and basal plasma membranes show differences in their lipid and protein composition; for example, caveolae, a membrane microdomain rich in sphingomyelin and glycosphingolipids, has been reported mainly in the basal membrane [14]. Naturally, many glycoproteins and proteoglycans, such as selectins and podocalyxin, occupy the apical/luminal domain to form attachment sites for immune cells [15], as well as complex entities, such as the glycocalyx for sensing blood flow and providing a sieving microenvironment [16]. Endocytic movement in ECs is also polarised. This is illustrated by the direction of transcytosis, which is determined by the distribution of target receptors, included in the endocytotic vesicle, between the apical and basal membranes of the cell; for example, the receptor for transferrin is localised on the apical side of brain ECs, in line with its role of iron import from the blood [17]. Moreover, ECs can secrete proteins and other factors in a polarised manner. For example, von Willebrand factor (VWF) exists in different multimeric states and can be released by ECs via three different secretory pathways, constitutive, basal, and regulated. VWF can be released apically or basally, based on its multimeric state, and the secretory pathway. This polarised secretion leads to different functions in ECs [18].

## 2. Polarity at Blood–Brain and Blood–Retinal Barriers

The blood–brain and the blood–retinal barriers (BBB/BRB) nearly completely separate the brain and the eyes from the rest of the body and protect them from the compositional fluctuations that occur in the blood in order to maintain homeostasis [19]. Highly specialised ECs operate in a very polarised environment, and thus make the BBB/BRB very suitable for polarity studies.

**Figure 2:**
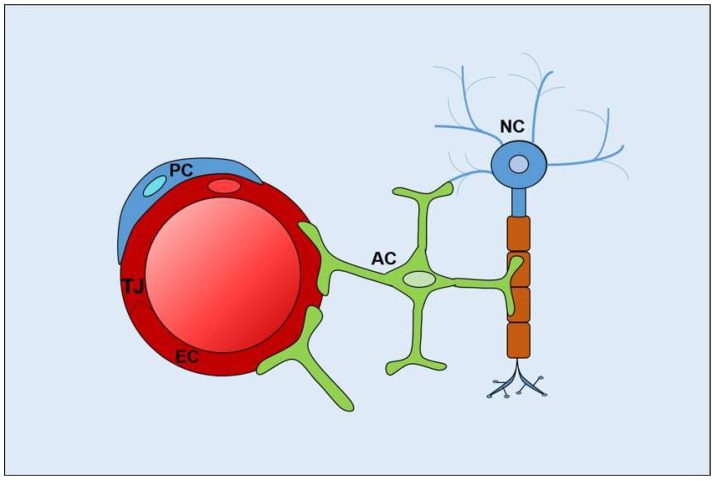
Cellular components of the neurovascular unit. EC, endothelial cells; TJ, tight junctions; PC, pericytes; AC, astrocytes; NC, neurons.

The abluminal surface of the neural endothelium is partially surrounded by a layer of pericytes, and together, these vascular cells lay on a basal membrane made up of extracellular matrix molecules. Astrocytes, a major glial cell type in the CNS, extend cellular processes that enclose the blood vessels as well as neuronal synapses and nodes of Ranvier. Pericytes and astrocytes contribute to establish and maintain blood–neural barriers, and together with ECs, neurons, and extracellular matrix components, form the neurovascular unit [19,20] (Figure 2).

The BBB/BRB shows low passive permeability to many polar nutrients, such as glucose and amino acids, which are essential for the nervous system homeostasis. The paracellular space between individual ECs is sealed by tight junctions (TJs), greatly limiting the movement of molecules and ions along the paracellular pathway (i.e., between cells) [21,22]. In addition, neural ECs lack fenestra and exhibit extremely low rates of pinocytosis/transcytosis, thus limiting the movement of molecules via a transcellular pathway (i.e., through the cell). The basic building blocks of TJs consist of a series of transmembrane molecules of the Claudin, MARVEL domain proteins, and junctional adhesion molecule (JAM) families [23]. These are linked to the cytoskeleton and other cellular domains through associated cytoplasmic adaptors, such as the zonula occludens (ZO) proteins. Importantly, apart from forming a tight regulated “gate” of the paracellular space, TJs also act as a “fence” within the membrane where they restrict proteins and lipids to either the apical or basal membrane domains, thus being instrumental for the formation and maintenance of the endothelial polarity [20]. Apical and basal membranes also differ in their lipid and glycoprotein composition, allowing ECs to secrete or transport soluble factors in a polarised manner [13]. Both membrane domains show a unique lipid composition with clear differences to each other and to the whole cell composition. For instance, phosphatidylcholine is enriched in the apical membrane, whereas sphingomyelin and glucosylceramide are predominantly found in the basal membrane [24].

Given the low permeability of the TJs, CNS ECs require a series of transporters to move ions, nutrients, and proteins from the blood to the brain. These transporters use a series of different mechanisms, including carrier-mediated transport, receptor-mediated transcytosis, and ion pumps [25]. Many of these transport proteins are polarised in their expression. Some are inserted into either the luminal or abluminal membrane only, like the P-glycoprotein [26] and Na^+^-dependent transporters [27], respectively, while others are inserted into both membranes of the ECs, like the OATP2 transporter [28].

Whilst TJs are instrumental in maintaining the boundaries for successful endothelial polarisation, complex molecular machineries regulate polarised secretion and sorting of membrane components, and thus, the establishment of functionally distinct apical and basal domains.

Brain ECs express all the main epithelial polarity complexes such as the Par, Scribble, and Crumbs proteins. The Par complex is localised at the TJ level, and it is involved in polarity during lumen formation [29]. TJs also bind proteins of the Crumbs complex, which stabilise intercellular junctions and decrease paracellular permeability [30]. Finally, as part of the Scribble complex, DLG1 enhances canonical WNT signalling in response to WNT7a, which is necessary for the development of the blood–brain barrier, whilst Scrib mediates planar cell polarity in ECs [31,32].

Complex regulators of brain endothelial polarity are the small GTPases RHOA, RAC, and CDC42, which are also regulated by shear stress [13]. RHOA is first inhibited and then activated by flow, and it increases BBB permeability in several ways. It intensifies the phosphorylation of the myosin light chain and contraction of actomyosin, pulling intercellular adhesions apart [33]; it enhances the phosphorylation of TJ proteins, which leads to their disintegration [34]; it redirects JAM-1 from TJs to the luminal surface, where it functions as a leukocyte adhesion molecule [35]. In contrast to the effects of RHOA, CDC42 enhances cell polarity and barrier properties, and promotes endothelial lumen formation [36].

Astrocytes and pericytes of the neurovascular unit also influence endothelial polarity. Astrocytes produce several factors to stabilise the barrier, such as angiotensin-II, glial cell-derived neurotrophic factor and angiopoietin 1. They also release the Sonic hedgehog factor, which activates the receptor Patched-1 on brain ECs, and stimulates the expression of the TJ components claudin-5 and occludin [37]. Pericytes are essential during the development of the blood–brain barrier, but also for its maintenance in adulthood [38]. Pericytes reduce paracellular permeability of adjacent ECs, by inducing the expression ZO-1 and occludin [39], but also their transcellular permeability, e.g. by regulating the expression of MFSD2A [40]. MFSD2A has also been identified as a sodium-dependent transporter for the ω3 fatty acid docosahexaenoic acid (DHA), present as the fatty acid chain in lysophosphatidylcholine (LPC) at the BBB/BRB [41,42]. This discovery led to the proposal of a novel mechanism for DHA/LPC from the blood to the brain parenchyma, whereby LPC is flipped from the outer to the inner layer of the plasma membrane by MFSD2A. This then allows apical to basal redistribution of DHA/LPC by diffusion within the inner leaflet of the plasma membrane, movement which is normally restricted by TJs in the outer leaflet [43,44]. Interestingly, LPC is also a pathologically important luminal leakage factor in BBB/BRB endothelium [45], pointing to a yet to be explored intimate relationship between leakage and lipid metabolism.

## 3. Vascular Endothelial Growth Factor Signalling in Endothelial Cells

Vascular endothelial growth factors (VEGFs) are dimeric glycoproteins that include VEGFA, VEGFB, VEGFC, VEGFD, and the placenta growth factor (PLGF). The structurally related VEGFE and VEGFF, from parapoxvirus and snake venom, respectively, can also bind to and activate cognate VEGF receptors. Alternative splicing generates a plethora of isoforms with different biological activities within each VEGF family. For instance, the VEGFA family includes VEGFA121, VEGFA145, VEGFA165, VEGFA189, and VEGFA206, each displaying different heparin binding and diffusion [46]. VEGFs bind to homo- or heterodimers composed of three single pass transmembrane protein tyrosine kinases, designated VEGFR1, -R2, and -R3. Their extracellular domains are organised into seven immunoglobulin-like folds that mediate ligand binding, interaction with co-receptors, and dimerisation. Intracellularly, they contain a juxtamembrane domain, a split tyrosine kinase domain, and a C-terminal tail containing tyrosine residues that undergo variable degrees of autophosphorylation and subsequent binding of SH2 domain-containing downstream effector proteins [47]. VEGFR1 displays binding for VEGFA, B, and PLGF; VEGFR2 for VEGFA (and VEGFE); and VEGFR3 for VEGFC and D [46]. Additionally, the neuropilin (NRP) family members NRP1 and NRP2, and heparin sulphate proteoglycans (HSPGs) act as functionally important co-receptors in the cellular response to VEGFs [48].

VEGFR1 can play a negative regulatory role in vascular biology as proved by its soluble form, named sFlt1, consisting of the extracellular domain of the receptor. sFlt1 binds VEGFA with high affinity, preventing the ligand from binding to full length VEGFR1 and VEGFR2. Moreover, VEGFR1−/− mice show uncontrolled EC proliferation and lumenless vessels, leading them to die during embryonic development [47]. VEGFR1 tyrosine phosphorylation sites have been determined, but downstream signalling remains poorly understood.

Much more is known of VEGFR2, and for more detailed accounts, we refer to excellent recent reviews [46,49,50]. Its homodimerisation and consequent kinase activation is assumed to mediate most of the VEGF effects on ECs. VEGFR2 plays essential roles in endothelial proliferation, differentiation, leakage, survival, and motility [51]. VEGFR2 phosphorylation on Y951 serves as a binding site for the T cell specific adaptor (TSAd) molecule, which binds and activates c-SRC at endothelial adherens junctions (AJs). SRC-mediated phosphorylation of the AJ protein VE-cad assists transient junction opening and the induction of leakage. VEGFR2 can also bind phospholipase Cγ (PLCγ) to induce a signalling pathway necessary for endothelial differentiation and proliferation during embryonic development. Several additional pathways appear to be induced by VEGF, like the AKT/phosphoinositide 3 kinase pathway, that may regulate endothelial survival [47]. VEGFR2 can be positively regulated through its interaction with the trimeric G proteins Gαq/Gα11, whilst its negative regulation is mediated by the SRC-homology phosphatase-1 (SHP1) and SHP2, which dephosphorylate the tyrosine residues [46]. Receptor activity is also downregulated by rapid degradation through the proteasome pathway, and through internalisation and degradation in the lysosomes after protein kinase C-dependent phosphorylation of the receptor C-terminal tail.

VEGFR2 receptor has also been found to be activated in non-canonical fashion, i.e., in the absence of VEGF. Non-VEGF ligands can bind and activate VEGFRs, and SRC family kinases can also phosphorylate and activate VEGFR2 in ligand-independent fashion [48,50]. Such ligand-independent transactivation of VEGFR2 operates in diabetes [52], or following cellular activation by LPC [45].

VEGFR3 binds VEGFC and VEGFD, and it was first discovered in lymphatic ECs, where it regulates lymphatic endothelial development and biology. Its expression has now also been shown in blood vascular ECs, where it can reinforce Notch signalling in tip cells, contributing to sprouting and vessel branching [53]. VEGFR3 can also be activated by integrin-mediated activation of SRC, contributing to survival and migration of ECs [54]. However, our focus here is on VEGFA, for which VEGFR3 signalling is not relevant.

Whilst there is considerable insight into the signalling of VEGFR homodimers, heterodimers undoubtedly play an important role in VEGF-mediated signalling, often with different functional outcomes [51]. Existence of both VEGFR1–VEGFR2, as well as VEGFR2–VEGFR3 heterodimers, has been proposed by signalling studies and computational modelling [55,56]. VEGFR2–VEGFR3 heterodimers are induced in vitro and in vivo on lymphatic ECs, in response to proteolytically processed VEGFC and VEGFD. VEGFR2–VEGFR3 heterodimers activate AKT signalling, whereas VEGFR3 homodimers induce ERK1/2 activation. This difference in downstream signalling is thought to be linked to failure of VEGFR2 to phosphorylate VEGFR3 on carboxyterminal tyrosines, which mediate binding of SHC and the consequent activation of the RAF-ERK1/2 pathway [50]. Much less is known about VEGFR1–VEGFR2 heterodimers. In most EC plasma membranes, VEGFR1 is up to tenfold less abundant than VEGFR2. Computational modelling of VEGF receptor subunit dimerisation predicts that, at these ratios, VEGFR1 is present as part of heterodimers with VEGFR2 [57]. Since no natural ligands are known to activate VEGFR1−VEGFR2 exclusively, Cudmore et al. have used a synthetic, heterodimeric ligand formed of a VEGFR2-specific monomer (VEGFE) and a VEGFR1-specific monomer (PLGF1), and demonstrated the existence of preassembled VEGFR1−VEGFR2 heterodimers in human primary ECs and animal tissues [58]. Stimulation with VEGFE/PLGF1 induces distinct tyrosine phosphorylation of VEGFR2 and downstream signalling, affecting EC migration, in vitro tube formation, and phosphorylation of endothelial nitric oxide synthase (eNOS). Subsequent NO release, known to feedback negatively on VEGF activity, points to a potential function of VEGFR1–VEGFR2 heterodimer signalling, namely to restrict VEGFR2 homodimer activity, which may be important to maintain EC homeostasis. Importantly, studies on VEGFR heterodimers indicate that they elicit downstream signalling distinct from that of the respective homodimers.

## 4. Polarised VEGFA Signalling at Blood–Neural Barriers

A major EC response to VEGFA, in particular at blood–neural barriers, is the induction of pathophysiological leakage. VEGFA can induce acute, sustained, and chronic leakage [59,60] and involves one or combinations of junctional remodelling, induction of fenestrae, vesiculo-vacuolar organelles (VVOs) or fluid-phase transcytosis. VVOs are clusters of connected vesicles, which form a membrane channel between luminal and abluminal surfaces of ECs. They occur within seconds after activation with VEGFA in vivo, and have been linked to acute permeability, in particular, in the tumour vasculature [61]. Fenestrae provide a similar direct opening through the ECs, albeit one with major restrictions on the size of permeating solutes. They are formed many minutes after the initial increase in permeability, and thus, consistent with the development of a chronic increase in vivo and in vitro [62]. Important to the discussion here is that neither of these two leakage pathways has been observed in the endothelium of blood–neural barriers [63]. Fluid-phase transcytosis has been observed following chronic VEGFA treatment in the retinal vasculature [64], but vesicle induction has, also, the potential to be induced in the acute phase of permeability [65]. Junction remodelling has been observed microscopically within several minutes and hours of VEGFA application, and may thus be involved in the sustained and chronic leakage response [66]. However, VEGFA also induces changes in electrical barrier properties within seconds in vitro, suggesting that ultrastructural changes of paracellular junctions may also occur in the acute phase of leakage [67]. Many VEGFA-induced leakage signalling pathways are activated within seconds of agonist exposure, and are thus compatible with regulating the acute phase. Prominent effector molecules are SRC, p38 [68,69], RHO GTPases, and RHO kinase (ROCK), as well as eNOS [70,71,72]. Indeed, pharmacological and genetic ablation has provided strong evidence for a key role of these molecules in VEGF-induced leakage. A good example for signalling towards sustained leakage is provided by the phosphorylation and disassembly of VE-cad, which is initiated within 5 min, but peaks at 30 min after addition of VEGFA to ECs in vitro [73]. Chronic leakage involves significant alterations of the vasculature with compositional changes in TJ proteins, such as the loss of occludin and claudins, or the loss of mural cells such as pericytes, and associated changes in gene expression [74].

**Figure 3:**
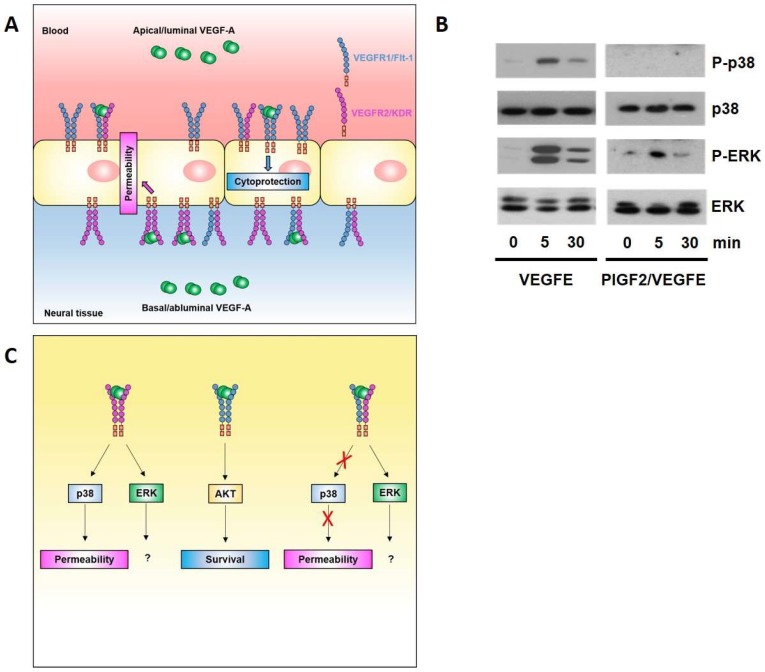
VEGFA signalling at the blood–brain barrier. (**A**) Brain ECs display sidedness in their response to VEGFA. The leakage-inducing response to VEGFA is exclusively mediated by the basal side of the endothelium and involves VEGFR2 homodimers and activation of the MAPK p38. In contrast, cytoprotective VEGFA signalling involves luminal VEGFR1 homodimers and AKT activation; (**B**) PLGF2/VEGFE induces phosphorylation of ERK, but not p38, indicating that leakage inducing p38 is a prerogative of VEGFR2 homodimers. Strep-tagged PLGF2 and His-tagged VEGFE were co-expressed in baculovirus, and purified by sequential affinity purification (details available on request). Primary rat brain ECs were stimulated with 50 ng/mL VEGFE or PLGF2/VEGFE, lysed, and processed for immunoblotting as described [67]; (**C**) Proposed signalling nodes for permeability and survival effector pathways triggered by VEGFR1 and R2 homo- and heterodimers.

The ECs of the BBB/BRB have, as detailed above, adapted to the highly polarised environment by developing highly selective and polarised transport machineries. Our group has recently reported that neural ECs also respond in a highly polarised manner to VEGFA [67] (Figure 3A). Indeed, leakage induction is restricted to abluminal VEGFA, whilst luminal VEGFA has a cytoprotective role. Mechanistically, this polarity in the response to VEGFA is due to polarised localisation of VEGFR1 and R2, with the former being predominant on the luminal face and the latter on the basal face of the neural endothelium. This results in the exclusive presence of VEGFR1 and R2 homodimers on the apical/luminal and basal/abluminal side, respectively. Stimulation with ligands selective for each of these homodimers, PLGF1 and VEGFE, shows that apical VEGFR1 homodimers mediate a cytoprotective response via AKT, whilst only basal VEGFR2 homodimers mediate a leakage response via p38.

Our data showed that polarised localisation of VEGFR1 and VEGFR2 is not absolute, and thus, implies that VEGFR1–R2 heterodimers also exist at both the apical and basal face of the endothelium. However, since apical VEGFA stimulation does not induce leakage, VEGF1–R2 heterodimers are unlikely to be involved in leakage signalling. Indeed, using a synthetic heterodimeric PlGF2–VEGFE(NZ2) ligand, we found that R1–R2 stimulation did not lead to p38 activation (Figure 3B), indicating that leakage inducing p38 is a prerogative of VEGFR2 homodimers, and thus, the basal face of the neural endothelium (Figure 3C). The functional consequences of ERK activation, which appear to be associated with all VEGFR2 conformer activation in the neural endothelium, still await elucidation.

## 5. Polarised Signalling at the BBB/BRB by Factors Other than VEGF

Our study on VEGF signalling was the first to put a functional and molecular framework to polarised paracrine signalling at the BBB/BRB [67]. However, other agonists also induce polarised signalling. Histamine induces leakage predominantly from the basal face of ECs, whereas lysophosphatidic acid does so much more strongly from the apical side [67,75]. For histamine, the sided response may be caused by differential expression and localisation of H1 and H2 receptors [76]. TGFβ/BMP signalling has been implicated in both vascular leakage and homeostasis [77,78], and may conceivably do so through a polarised endothelial response to different ligands. LPC, a circulating lipid mobilised by a phospholipase A2 from oxidised low density lipoprotein is also a potent leakage factor, implicated in diabetes-induced retinal leakage [45]. It does so exclusively from the luminal EC side in a process involving transactivation of VEGFR2 (which in its leakage-inducing form resides on the abluminal side), indicating that not only polarised signalling modules exist on both faces of the neural endothelium, but also points to communication between apical and basal signalling, which presumably integrate endothelial behaviour at the border between two biophysically and biochemically very distinct environments.

## 6. Conclusions and Future Perspectives

Endothelial polarity is clearly an essential trait that adapts the vasculature to its diametrically different surroundings. Its importance for the barrier function in the neural vasculature has been recognised for some time, with the polarisation of transport systems studied extensively in many laboratories. It is in this vascular bed that VEGFA signalling polarisation is most readily discernible. Currently, it is not known if similar polarisation also exists in other vascular beds. Unlike the brain, the lung vasculature leaks in response to circulating (i.e., luminal) VEGFA [67]. However, this may not point to an absence of abluminal VEGFR2 polarisation, since in this more leaky endothelium, VEGFA may readily diffuse to the basal membrane and trigger a leakage response there. Indeed, in their permeability response, human umbilical vein ECs are much more sensitive to basal than apical VEGFA (Silvia Dragoni and Patric Turowski, unpublished observation), suggesting some degree of receptor polarisation in these non-neural ECs. Only detailed localisation studies of VEGF receptors will fully define the polarisation of the response to VEGFA in non-neural ECs.

Thus, ECs have adapted to environmental polarity by polarising and specifying signalling pathways, most of which remain to be described. For VEGFA, the molecular strategy for polarised signalling involves the selective localisation of response specific receptor at either the luminal or abluminal face of the endothelium. However, strategies of similar distribution of receptors but uneven association of signalling adaptors are also conceivable. Caveolae, which contain many signalling complexes and can relocate [62], may constitute another way to adapt the EC to its polarised environment.

With respect to VEGF signalling, our understanding of the molecular mechanisms and function of its polarity has taken a significant step forward, but some important questions remain. First, the sorting processes that ascertain an uneven distribution of the receptors on the luminal and abluminal faces of the ECs need to be elucidated. It is likely that this involves processes akin to those described in epithelial cells [79]. In that regard, it is also unclear how defective sorting of VEGF receptors affects endothelial physiology and if this is relevant for pathophysiology. Lastly, it is likely that alternate receptor conformations and co-receptors will influence and fine-tune polarised VEGFA signalling.
